# First two fungemia cases caused by *Candida haemulonii var. vulnera* in China with emerged antifungal resistance

**DOI:** 10.3389/fmicb.2022.1036351

**Published:** 2022-11-10

**Authors:** Xin-Fei Chen, Xin Hou, Han Zhang, Xin-Miao Jia, Li-Ping Ning, Wei Cao, Xin Fan, Jing-Jing Huang, Wen-Hang Yang, Ge Zhang, Jing-Jia Zhang, Wei Kang, Meng Xiao, Ying-Chun Xu

**Affiliations:** ^1^Department of Laboratory Medicine, State Key Laboratory of Complex Severe and Rare Diseases, Peking Union Medical College Hospital, Chinese Academy of Medical Sciences and Peking Union Medical College, Beijing, China; ^2^Graduate School, Chinese Academy of Medical Sciences and Peking Union Medical College, Beijing, China; ^3^Beijing Key Laboratory for Mechanisms Research and Precision Diagnosis of Invasive Fungal Diseases (BZ0447), Beijing, China; ^4^Department of Laboratory Medicine, Peking University Third Hospital, Beijing, China; ^5^Medical Research Center, Peking Union Medical College Hospital, Chinese Academy of Medical Sciences and Peking Union Medical College, Beijing, China; ^6^Department of Laboratory Medicine, No.908 Hospital of Joint Logistics Support Force, Nanchang, Jiangxi, China; ^7^Department of Laboratory Medicine, The Second Xiangya Hospital, Central South University, Changsha, Hubei, China; ^8^Department of Infectious Diseases and Clinical Microbiology, Beijing Institute of Respiratory Medicine, Beijing Chao-Yang Hospital, Capital Medical University, Beijing, China

**Keywords:** *Candida haemulonii var*. *vulnera*, antifungal susceptibility, *ERG11*, *FUR1*, whole-genome sequence, drug resistant mechanisms

## Abstract

*Candida haemulonii var. vulnera* is a rare variant of *C. haemulonii*, which has been previously reported to cause human infections. Owing to the close kinship between *C. haemulonii* sensu stricto and *C. haemulonii var. vulnera*, accurate identification of *C. haemulonii var. vulnera* relied on DNA sequencing assay targeting, for example, rDNA internal transcribed spacer (ITS) region. In this work, two strains of *C. haemulonii var. vulnera* were collected from the China Hospital Invasive Fungal Surveillance Net (CHIF-NET). The identification capacity of three matrix-assisted laser desorption/ionization time-of-flight mass spectrometry (MALDI-TOF MS) and VITEK 2 YST ID biochemical methods were evaluated against ITS sequencing. In addition, antifungal susceptibility testing was performed using Sensititre YeastOne. Moreover, we comprehensively screened drug-resistant related genes by whole-genome sequencing. The two strains were not correctly identified to species variant level using MALDI-TOF MS and YST ID cards. Both strains were resistant to amphotericin B (minimum inhibitory concentration [MIC] > 2 μg/ml). Moreover, strain F4564 and F4584 exhibited high MIC to fluconazole (>256 μg/ml) and 5-flucytosine (>64 μg/ml), respectively, which were supposed to result from key amino acid substitutions Y132F and G307A in Erg11p and V58fs and G60K substitutions in Fur1p. The rare species *C. haemulonii var. vulnera* has emerged in China, and such drug-resistant fungal species that can cause invasive diseases require further close attention.

## Introduction

*Candida haemulonii var. vulnera* belongs to the *C. haemulonii* species complex, along with *C. haemulonii* sensu stricto and *C. duobushaemulonii* (Gade et al., 2020; Rodrigues et al., 2021; Ramos et al., 2022). Generally, *C. haemulonii* species complex isolates display high multidrug-resistant rates and transmission properties, which have attracted increased attention (Gade et al., 2020).

In 2012, *C. haemulonii var. vulnera* was reported for the first time by Cendejas-Bueno et al. They found four strains with low rDNA internal transcribed spacer (ITS) sequence identity to *C. haemulonii* sensu stricto type strain (∼96%) (Cendejas-Bueno et al., 2012). Infections caused by *C. haemulonii var. vulnera* were later reported in Brazil, India, Argentina, and Peru (de Almeida et al., 2016; Kumar et al., 2016; Isla et al., 2017; Pérez-Lazo et al., 2021; Ramos et al., 2022). In addition, by retesting a set of previously collected strains, Rodrigues et al. found a *C. haemulonii var. vulnera* strain isolated in 2009, which became the earliest strain of the species variant discovered till now and its genome sequence was elucidated (Rodrigues et al., 2020). Moreover, antifungal resistance to azoles, echinocandins, and amphotericin B has been reported in *C. haemulonii var. vulnera* isolates (Cendejas-Bueno et al., 2012; Isla et al., 2017; Ramos et al., 2022).

In this study, we reported two fungemia cases caused by *Candida haemulonii var. vulnera* found from China Hospital Invasive Fungal Surveillance Net (CHIF-NET) study. Clinical characters, identification capacity of Vitek YST card and three matrix-assisted laser desorption/ionization time-of-flight mass spectrometry (MALDI-TOF MS) systems, isolates’ antifungal susceptibility phenotypes, and potential resistant mechanisms were illustrated. To our best knowledge, these were the first *Candida haemulonii var. vulnera* infection cases reported in China, including the first 5-flucytocine resistant strain discovered globally.

## Materials and methods

### Ethics statement

This study was approved by the Human Research Ethics Committee of the Peking Union Medical College Hospital (No. S-263). Written informed consent was obtained from all patients who participated in this study, which aimed to culture and study the isolates obtained from the patients.

### Microorganisms and identification

**FIGURE 1:**
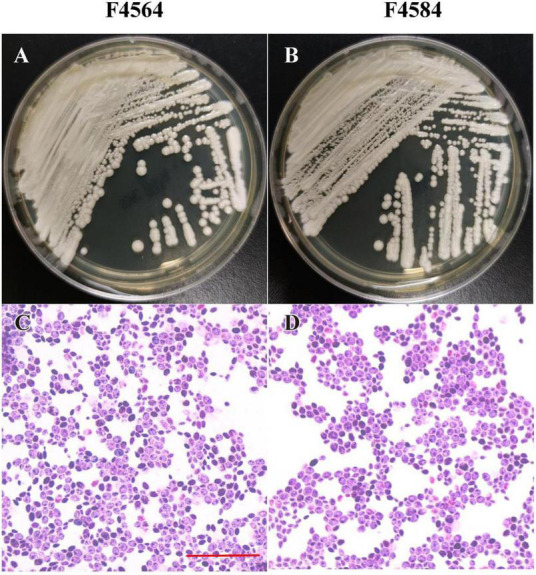
Phenotypic characteristics of two *C*. *haemulonii var. vulnera* isolates on Sabouraud dextrose agar. The plate contains colonies with typical appearance of circular, smooth, and moist **(A,B)**. Microscopic examination showed budding yeast cells with Gram’s staining **(C,D)**. Scale bar = 10 μm **(C)**.

From 2010 to 2017 (Table 1), two *C. haemulonii var. vulnera* isolates were collected from different hospitals in two provinces from the CHIF-NET study. The colony morphology of two strains is smooth, moist, and circular, which is the typical appearance of *Candida* species. Gram-stained microscopy showed budding yeast cells (Figure 1). The isolates were identified using Autof MS 1000 (Autobio, Zhengzhou, China), Smart MS (DL, Zhuhai, China), and Vitek MS (bioMérieux, Marcy l’Étoile, France) MALDI-TOF MS systems, in addition to Vitek 2 YST ID Card using VITEK 2 (9.02 version, bioMérieux, Marcy-l’Etoile, France) following the manufacturer’s instructions. Primers ITS1 and ITS4 (Hou et al., 2016) were used for ITS amplification and sequencing, and Sanger sequencing was performed using ABI 3730XL DNA analyzer (Thermo Fisher Scientific, Cleveland, OH, USA). A phylogenetic tree of the ITS sequences was constructed using Mega X based on 1000 bootstrap replicates using the maximum likelihood method (Kumar et al., 2018).

**TABLE 1:** Information for two *Candida haemulonii var. vulnera* isolates identified in this study.

Strain	F4564	F4584
**General information**		
Age/Gender	88/Female	12/Male
Year of isolation	2015	2016
Source of isolate	Blood	Blood
Clinical diagnosis	Pulmonary infection	Abdominal infection
Ward	ICU	General surgery
Location	Nanchang, China	Changsha, China
**Identification (identity/score/confidence value)**		
ITS sequencing	*C. haemulonii var. vulnera* (100%)	*C. haemulonii var. vulnera* (100%)
Vitek 2 Compact	Low discrimination	*C. haemulonii* (94%)
Vitek MS	*C. haemulonii* (99.9)	*C. haemulonii* (99.9)
Autof-MS 1000	*C. haemulonii* (9.505)	*C. haemulonii* (9.516)
Smart MS	No identification	No identification
**Antifungal susceptibility (μg/ml)**		
Fluconazole	>256	16
Voriconazole	2	0.12
Itraconazole	0.25	0.25
Posaconazole	0.25	0.12
5-Flucytosine	<0.06	>64
Anidulafungin	0.12	0.12
Micafungin	0.12	0.12
Caspofungin	0.12	0.12
Amphotericin B	4	4
**Potential resistance mechanisms**		
Erg11p	Y132F and G307A	WT
Fur1p	WT	V58fs and G60K

WT: wild type; fs: frameshift mutation; ICU: intensive care unit.

### DNA extraction and whole-genome sequencing

The whole genomic DNA of *C. haemulonii var. vulnera* was extracted as previously reported (Huang et al., 2022). The 350-bp DNA library was constructed using NEBNext^®^ Ultra™, following the manufacturer’s instructions. Library integrity was assessed using an Agilent 2100 Bioanalyzer (Agilent Technologies). Sequencing was performed on an Illumina NovaSeq using the PE150 strategy (Beijing Novogene Bioinformatics Technology Co., Ltd.). The Illumina reads generated in this study were obtained from the National Center for Biotechnology Information (NCBI) under BioProject PRJNA890168.

### Genome analysis

The *C. haemulonii var. vulnera* K1 (GenBank accession number GCA_012184645.1) genome was concatenated and used as a reference genome for read mapping. BWA 0.5.9, SAMtools, and bcftools 0.1.19 (Li and Durbin, 2009; Li et al., 2009) were used for single-nucleotide polymorphism (SNP) and insertion/deletion (indel) analysis, and snpEff 4.3 was used for SNP and indel function annotations (Cingolani et al., 2012). Mutations on antifungal-resistant related genes, including *ERG11* and *TAC1*b for azoles, *FUR1* for 5-flucytosine, *FKS1* and *FKS2* for echinocandins, *ERG3* and *ERG6* for amphotericin B, and *ERG4* for other ergosterol pathway genes, were analyzed in detail (Arendrup and Patterson, 2017; Berkow and Lockhart, 2017).

### Antifungal susceptibility testing

Antifungal susceptibility testing was performed using Sensititre YeastOne YO10 methodology (Thermo Scientific, Cleveland, OH, USA). *Candida krusei* ATCC 6258 and *Candida parapsilosis* ATCC 22019 were used as quality controls. As clinical breakpoints or epidemiological cutoff values against *C. haemulonii var. vulnera* have not been established, we used interpretation criteria for *Candida* species referring to CLSI M27-S3 guidelines (CLSI, 2008), including MIC value of ≥32 μg/mL as resistance to fluconazole ≥32 μg/mL as resistance to 5-flucytosine. In addition, MIC of ≥2 μg/mL was used for interpreting “resistance” to amphotericin B (Pfaller et al., 2012).

### Review of *Candida haemulonii var. vulnera* cases reported

A literature review of previously reported *C. haemulonii var. vulnera* infections was done. A literature search was performed on 28 August 2022, using the following three databases: PubMed^1^, Web of Science^2^, and Embase^3^. The terms “*Candida haemulonii var. vulnera*” were entered in the category of “Title/Abstract” in the PubMed Advanced Search Builder, and “TS (*Candida haemulonii var. vulnera*)” was entered into the Web of Science database. The search in Embase was conducted in the advanced search area, including the terms “*Candida haemulonii var. vulnera*’: ab,ti.”. All hits were further screened manually to find out all infection cases with antifungal susceptibility reports.

## Results

### Isolate information

The first case was an 88-year-old male patient who was admitted to the intensive care unit (ICU) with clinical diagnosis of pulmonary infection. F4564 was isolated from peripheral blood of this patient and initially misidentified as “*Candida krusei*” using CHROMagar chromogenic medium at local laboratory (Table 1). The second case was a 12-year-old boy who was admitted to the general surgical ward, and clinical diagnosis was abdominal infection. F4584 was isolated from peripheral blood of this patient and identified as “*Candida spp.*” using CHROMagar chromogenic medium initially (Table 1).

### Identification of *Candida. haemulonii var. vulnera* using ITS sequencing, MALDI-TOF MS, and Vitek 2

**FIGURE 2:**
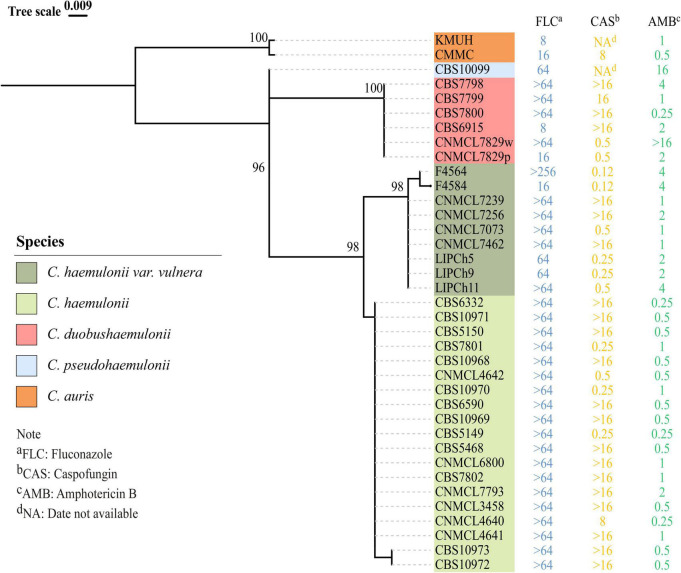
Phylogenetic tree for rDNA ITS region of previously reported *Candida haemulonii*, *C*. *duobushaemulonii*, *C*. *pseudohaemulonii*, *C*. *auris*, and *C*. *haemulonii var. vulnera*, including two *C*. *haemulonii var*. *vulnera* clinical strains identified in this study. Phylogenetic tree was constructed using Mega X with 1000 bootstrap replicates based on the maximum likelihood method.

The ITS sequences of the isolates exhibited 100% identity with the corresponding ITS sequences of the reference *C. haemulonii var. vulnera* CBS12439^T^ isolates (GenBank accession number: JX459686.1). Furthermore, both clinical isolates were identified as *C. haemulonii* by the Autof MS 1000 and Vitek MS, but with “no identification” results by Smart MS. While using the Vitek 2 Compact system, one strain was identified with low discrimination and the other was identified as *C. haemulonii* (score = 94%) (Table 1). In phylogenetic tree generated by *C. haemulonii* complex, *C. auris*, and *C. pseudohaemulonii* ITS sequences, F4564 and F4584 were in the branch of *C. haemulonii var. vulnera* (Figure 2).

### Genome analysis

We sequenced genomes of F4564 and F4584, and their total genome sizes were 13.51 Mb and 13.26 Mb, respectively, with an average GC content of 45% (N50 was 346,464 bp and 283,647 bp, and a number of assembled contigs were 270 and 262, comprising 95.7% and 95.6% of data according to BUSCO analysis, respectively).

### Antifungal susceptibility

MIC values obtained for the nine antifungal agents against *C. haemulonii var. vulnera* isolates are shown in Table 1. F4564 was resistant to fluconazole with an MIC of >256 μg/ml. In addition, F4564 was classified as susceptible dose-dependent to voriconazole (2 μg/ml) and itraconazole (0.25 μg/ml). F4684 was resistant to 5-flucytosine (>64 μg/ml) and susceptible dose-dependent to itraconazole. In addition, both isolates had high MIC values of 4 μg/ml for amphotericin B, while neither strain was non-susceptible to posaconazole, caspofungin, anidulafungin, and micafungin (Table 1).

### Potential genomic variations contributed to antifungal resistance

Compared with the reference genome K1, which is from an isolate susceptible to all antifungals except for amphotericin B, we found that F4564 has two previously reported key amino acid substitutions (Y132F and G307A) in Erg11p. Interestingly, we discovered two novel mutations in Fur1p (V58fs and G60K) in strain F4584 with high 5-flucytosine MIC. However, we did not find any key variation in sterol metabolism-related genes like *ERG2*, *ERG3*, and *ERG6* that may result in amphotericin B resistance. In addition, all strains were susceptible to echinocandins and did not carry substitutions in Fks1p or Fks2p.

### Literature review

We found eight articles in all reported antifungal susceptibility of *C. haemulonii var. vulnera* isolates. A number of strains exhibit high MICs to fluconazole or even all azoles (Cendejas-Bueno et al., 2012), but there was not any investigation on related resistance mechanisms. In addition, one study reported the emergence of pan-echinocandin-resistant *C. haemulonii var. vulnera* strains (Cendejas-Bueno et al., 2012). To date, there have not been any reports describing 5-flucytosine-resistant *C. haemulonii var. vulnera* strain (Table 2).

**TABLE 2:** Overview of published reports on antifungal susceptibility profiles of *Candida haemulonii var. vulnera.*

No. isolates [Reference]	Strain or isolate	Antifungals (μg/ml)									Method
			
		FLU	VRC	ITC	POS	MCF	CAS	ANF	AMB	5FC	
***N* = 7 (Ramos et al., 2022)**											
MIC	LIPCh14	>64	>16	>16	>16	0.06	0.25	0.125	2	0.25	CLSI
	LIPCh18	>64	>16	>16	>16	0.125	1	0.125	4	0.25	
	LIPCh20	>64	>16	>16	>16	0.125	1	0.25	8	0.25	
	LIPCh21	>64	>16	>16	>16	0.125	1	0.06	4	0.25	
	LIPCh24	64	0.25	0.25	0.125	0.125	1	0.06	16	0.25	
	LIPCh25	>64	>16	>16	>16	0.06	0.5	0.06	4	0.25	
	LIPCh37	>64	>16	>16	>16	0.06	0.5	0.06	8	<0.125	
***N* = 3 (Ramos et al., 2015)**											
MIC	LIPCh5	64	0.5	0.5	ND	ND	0.25	ND	2	ND	CLSI
	LIPCh9	64	16	8	ND	ND	0.25	ND	2	ND	
	LIPCh11	>64	>16	16	ND	ND	0.5	ND	4	ND	
***N* = 2 (Rodrigues et al., 2020)**											
MIC	K1	8	0.25	0.5	0.25	0.12	0.25	0.06	8	< 0.06	YeastOne
	K2	16	0.25	0.5	0.25	0.12	0.25	0.06	2	< 0.06	
***N* = 4 (Cendejas-Bueno et al., 2012)**											
MIC	CNM-CL7239	>64	>8	>8	> 8	>16	> 16	>16	1	< 0.12	EUCAST
	CNM-CL7256	>64	>8	>8	> 8	0.12	> 16	0.06	2	0.5	
	CNM-CL7073	>64	>8	>8	> 8	0.12	0.5	0.06	1	0.25	
	CNM-CL7462	>64	>8	>8	> 8	0.06	> 16	< 0.03	1	0.5	
***N* = 1 (Pérez-Lazo et al., 2021)**											
MIC		>64	ND	ND	ND	ND	ND	0.06	1	ND	CLSI
***N* = 8 (de Almeida et al., 2016)**											
GM		17.4	11.53	ND	ND	ND	0.26	0.016	1	ND	CLSI
MIC_90_		64	16	ND	ND	ND	0.5	0.03	2	ND	
MIC range		2- > 64	0.125 – > 16	ND	ND	ND	0.125-0.5	< 0.015-0.03	0.5-2	ND	
***N* = 5 (Isla et al., 2017)**											
Mode		2	0.03	0.5	0.06	0.12	0.12	ND	4	0.12	EUCAST
GM		8	0.24	0.33	0.07	0.14	0.12	ND	3.48	0.12	
MIC range		2-128	0.03-8	0.06-1	0.0-0.5	0.12-0.25	0.006-0.25	ND	2-8	0.12	
MIC_50_		4	0.1	0.5	0.06	0.12	0.12	ND	4	0.12	
MIC_90_		128	8	1	0.25	0.25	0.25	ND	8	0.12	
***N* = 3 (Rodrigues et al., 2021)**											
MIC range		16-32	0.06-0.12	ND	ND	0.06-0.12	0.06-0.12	0.015	ND	0.25-1	CLSI

FLC: Fluconazole; VRC: Voriconazole; ITC: Itraconazole; POS: Posaconazole; CAS: Caspofungin; ANF: Anidulafungin; MCF: Micafungin; AMB: Amphotericin B; 5FC: 5-flucytosine; MIC: minimum inhibitory concentration; GM: geometric mean; MIC_50_: minimum inhibitory concentration able to inhibit 50% of all isolates tested; MIC_90_: minimum inhibitory concentration able to inhibit 90% of all isolates tested.

## Discussion

*Candida haemulonii var. vulnera* was identified ever-first by ITS sequencing (Cendejas-Bueno et al., 2012). Although it has been involved in identification database of latest Vitek 2 YST ID system, the results from the current study and Rodrigues et al. (2020) still found YST ID card was not able to achieve a reliable identification between *C. haemulonii* sensu stricto and *C. haemulonii var. vulnera*. Furthermore, none of the current available MALDI-TOF MS systems could accurately identify *C. haemulonii var. vulnera* (Kathuria et al., 2015; Grenfell et al., 2016; Rodrigues et al., 2020), though Grenfell *et al.* described that some discriminatory protein peaks may be able to differentiate *C. haemulonii* sensu stricto and *C. haemulonii var. vulnera* in using FlexAnalysis and ClinProTools to analysis MALDI-TOF MS results (Grenfell et al., 2016). Therefore, DNA sequencing remained the only applicable methods for the identification of *C. haemulonii var. vulnera*. Interestingly, Cendejas-Bueno reported that *C. haemulonii* sensu stricto and *C. haemulonii var. vulnera* can be separated by ITS sequences, but these two species have identical *RPB1*, *RPB2*, and D1/D2 sequences. Therefore, these strategies could not be used for the identification of these two species (Cendejas-Bueno et al., 2012). To date, isolation of *C. haemulonii var. vulnera* has only been reported in Brazil, Argentina, and Peru. Due to the above-mentioned limitations of commercial identification systems, we reidentified all *C. haemulonii* complex isolates collected in CHIF-NET study by ITS sequencing and finally found two *C. haemulonii var. vulnera* strains among >80 cases.

Previously, fluconazole- and amphotericin B-resistant *C. haemulonii var. vulnera* have been discovered (Cendejas-Bueno et al., 2012; Ramos et al., 2015; de Almeida et al., 2016). Like findings in previous reports, one of the isolates we discovered was also resistant to fluconazole and amphotericin B. However, we also found a 5-flucytosine-resistant strain, and it is also cross-resistant to amphotericin B. Although 5-flucytosine resistance has been recognized in *C. haemulonii* sensu stricto and *C. duobushaemulonii* in China and other regions (Kathuria et al., 2015; Hou et al., 2016), this is the first 5-flucytosine-resistant *C. haemulonii var. vulnera* case characterized to date. Of note, both strains we discovered remained susceptible to all echinocandins.

Compared with *C. haemulonii* sensu stricto and *C. duobushaemulonii* that have been well characterized, there are very few studies on resistant mechanisms of *C. haemulonii var. vulnera*. Key amino acid substitutions in Erg11p and Fur1p have been noted as major reasons contributing to azole and 5-flucytosine resistance, respectively, in other *C. haemulonii* complex species (Gade et al., 2020) and Chen et al. (2022). In this study, it was found that the fluconazole-resistant *C. haemulonii var. vulnera* isolate carried Y132F and G307A mutations in Erg11p. Erg11p Y132F substitutions have been reported in a broad range of fluconazole-resistant *Candida* species including *C. albicans*, *C. tropicalis*, and *C. haemulonii* (Fan et al., 2019; Warrilow et al., 2019; Gade et al., 2020), while G307A has been reported in *C. parapsilosis* (Arastehfar et al., 2020; Binder et al., 2020). While in our 5-flucytosine-resistant *C. haemulonii var. vulnera* strain, a frameshift V58fs and a mutation G60K were found in Fur1p, which has not been recovered in any other *Candida* strains to our best knowledge. Literature review showed a high proportion of *C. haemulonii var. vulnera* isolates (13/17, 76.5%) were with reduced susceptibility to amphotericin B (≥2 μg/ml). However, resistant mechanisms to amphotericin B were not well understood, and in this study, we also failed to found any mutations in key genes may potentially contributed to amphotericin B resistance. As a major limitation, our study did not collect detailed medical records of these two cases; therefore, we were not able to further analyze the source of infections or assessing clinical risk factors of this species.

In conclusion, there is a potential threat posed by *C. haemulonii var. vulnera*, a highly antifungal-resistant fungal species. Resistance to fluconazole and 5-flucytosine in *C. haemulonii var. vulner*a was supposed to be resulted from variations in Erg11p and Fur1p, respectively. Although invasive infection with *C. haemulonii var. vulnera* remained rare, further monitoring of this specie is still warranted.

## Data availability statement

The datasets presented in this study can be found in the online repositories. The names of the repository/repositories and accession numbers(s) can be found in the manuscript.

## Author contributions

X-FC, XH, HZ, and X-MJ conceived and designed the experiments. L-PN and WC provided the isolates. X-FC, HZ, X-MJ, XF, XH, J-JH, W-HY, GZ, J-JZ, and WK performed experiments. X-FC and HZ analyzed the data and wrote the manuscript. MX, XH, and Y-CX revised the manuscript. All authors contributed to the manuscript and approved the submitted version.

## Footnotes

1
https://pubmed.ncbi.nlm.nih.gov

2
https://webofknowledge.com

3
https://www.embase.com
